# Iterative Image Reconstruction for Sparse-View CT Using Normal-Dose Image Induced Total Variation Prior

**DOI:** 10.1371/journal.pone.0079709

**Published:** 2013-11-18

**Authors:** Jing Huang, Yunwan Zhang, Jianhua Ma, Dong Zeng, Zhaoying Bian, Shanzhou Niu, Qianjin Feng, Zhengrong Liang, Wufan Chen

**Affiliations:** 1 School of Biomedical Engineering, Southern Medical University, Guangzhou, China; 2 Department of Radiology, State University of New York, Stony Brook, New York, United States of America; Virginia Tech, United States of America

## Abstract

X-ray computed tomography (CT) iterative image reconstruction from sparse-view projection data has been an important research topic for radiation reduction in clinic. In this paper, to relieve the requirement of misalignment reduction operation of the prior image constrained compressed sensing (PICCS) approach introduced by Chen *et al*, we present an iterative image reconstruction approach for sparse-view CT using a normal-dose image induced total variation (ndiTV) prior. The associative objective function of the present approach is constructed under the penalized weighed least-square (PWLS) criteria, which contains two terms, *i.e.*, the weighted least-square (WLS) fidelity and the ndiTV prior, and is referred to as “PWLS-ndiTV”. Specifically, the WLS fidelity term is built based on an accurate relationship between the variance and mean of projection data in the presence of electronic background noise. The ndiTV prior term is designed to reduce the influence of the misalignment between the desired- and prior- image by using a normal-dose image induced non-local means (ndiNLM) filter. Subsequently, a modified steepest descent algorithm is adopted to minimize the associative objective function. Experimental results on two different digital phantoms and an anthropomorphic torso phantom show that the present PWLS-ndiTV approach for sparse-view CT image reconstruction can achieve noticeable gains over the existing similar approaches in terms of noise reduction, resolution-noise tradeoff, and low-contrast object detection.

## Introduction

Radiation risk in x-ray computed tomography (CT) examinations has caused significant concerns to patients due to the negative effects of x-ray exposure [1], [2]. To reduce the radiation dose of CT scans, many investigations have been performed including the hardware-based scanning protocols [3], [4], [5] and software-based image reconstruction techniques [6], [7], [8]. It is known that lowering the milliampere-seconds (mAs) [9], [10], [11], [12], [13] or reducing the number of projections per rotation around the body [14], [15], [16], [17], [18], [19], [20] is an important means for reducing radiation dose. However, the associative image quality would be unavoidably deteriorated due to the noisy or sparse-view measurements if no adequate noise control is applied in image reconstruction. In this study, we are focusing on CT image reconstruction from the reduced number of projection per rotation or sparse-view projection data.

In modern CT systems, several hundred or even over a thousand of projection per rotation are acquired for image reconstruction [21]. Theoretically, cutting half of the projections would reduce radiation dose by a half. However, due to insufficient sampling with sparse-view measurements, conventional filtered back-projection (FBP) approach cannot yield high-diagnostic image quality. To address this problem, Sidky *et al* [22] formulated an innovative algorithm based on projection onto convex sets (POCS), called TV-POCS, by adapting total variation (TV) minimization of the desired-image with piecewise constant assumption. As an updating TV-POCS algorithm, an adaptive-steepest-descent based POCS (ASD-POCS) algorithm [16] was proposed for minimizing TV with an improved performance against cone-beam artifacts in sparse-view CT image reconstruction. Due to the assumption of isotropic edge property within TV minimization, the related algorithms often suffer from over-smoothing effects. Hence, the weighted-TV as an extension of the original one were proposed recently to address the aforementioned issue in sparse-view CT image reconstruction [20], [23].

In clinic, repeated scans during a treatment course are often required in specific applications including dynamic CT angiography, perfusion CT, and CT-guided interventional procedures [2], [24]. In these conditions, the previous normal-dose scanned data can be referred to as “normal-dose prior image” aiming to facilitate subsequent CT image reconstructions with sparse-view projection measurements [13], [15], [25], [26], [27]. For example, Chen *et al* proposed a prior image constrained compressed sensing (PICCS) approach for sparse-view CT image reconstruction by incorporating a prior image [15]. The PICCS algorithm has been extensively tested on patient and animal data with sound results in different applications [25], [28]. However, the PICCS algorithm assumes that the misalignment between the desired- and prior- image cannot be significant. Meanwhile, this assumption cannot usually be met in practice. A typical example is in time-resolved CT or four-dimensional CBCT (4D-CBCT) imaging, the patient position is frequently changed from one scan to another within the time-series data acquirement [29]. Additionally, in perfusion CT, vessels and perfused tissues would change their attenuation properties after the intravascular contrast agent mixed with blood [30]. In these cases, the performance of PICCS would be inevitably influenced by the mismatched tissues between the desired- and prior- image. To address this problem, a misalignment reduction operation could be executed in the implementation of the original PICCS approach [31], [32], [33]. For example, Nett *et al* proposed a modified PICCS approach with combining a registration step to minimize the misalignment between the prior- and desired- image [31]. Meanwhile, the performance of those approaches would heavily depend on the accuracy of image registration operations.

In this paper, based on the recent studies about sparse-view and low-dose CT image reconstructions [11], [12], [13], [28], [34], [35], [36], [37], [38], [39], we propose a normal-dose image induced total variation prior (ndiTV) under the penalized weighted least-square (PWLS) criteria [10], which is referred to “PWLS-ndiTV” for simplicity, aiming to relieve the requirement of misalignment reduction operation of the PICCS algorithm. The novelty of the present PWLS-ndiTV approach is twofold. First, the weighted least-square (WLS) fidelity term in the objective function of PWLS-ndiTV considers an accurate relationship between the variance and mean of projection data in the presence of electronic background noise, which explores the accurate statistical properties of CT projection data. Second, the ndiTV prior term is designed to reduce the influence of the misalignment between the desired- and prior- image by using a normal-dose image induced non-local means (ndiNLM) filter. Qualitative and quantitative evaluations for CT image reconstruction from sparse-view projection data were carried out on two digital phantoms and an anthropomorphic torso phantom in terms of noise reduction, resolution-noise tradeoff, and low-contrast object detection.

The remaining of the paper is organized as follows. Section Methods describes the PWLS criteria for CT image reconstruction, and the ndiTV prior and the associated PWLS-ndiTV image reconstruction algorithm are presented in detail. Moreover, the experimental setup and evaluation metrics are also outlined in this section. In Section Results, the evaluation results are reported, followed by Sections Discussion and Conclusion, respectively.

## Methods

### PWLS criteria for CT image reconstruction

Mathematically, the PWLS criterion for CT image reconstruction can be rewritten as follows [10], [13], [40]:
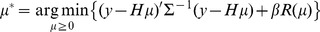
(1)where 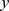 represents the obtained sinogram data (the projections after system calibration and logarithm transformation), *i.e.*, 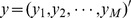, 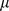 is the vector of attenuation coefficients to be estimated, *i.e.*, 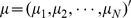, where “
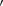
” denotes the matrix transpose. The operator 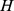 represents the system matrix with the size of 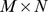. The element of 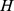 denotes the length of intersection of projection ray 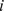 with pixel 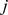 where the associated elements can be pre-calculated by using a fast ray-tracing technique [41]. 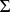 is a diagonal matrix with the 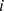
th element of 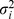 which is the variance of sinogram data 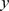 at bin 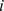. 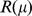 denotes a prior term. 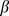 is a hyper-parameter for controlling the strength of prior term as a penalty. The goal for CT image reconstruction is to estimate the attenuation coefficients 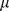 from the measurement 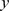 with 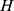.

Based on our previous works [11], [42], in this study, the variance of 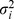 is determined by the following mean-variance relationship:
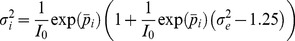
(2)where 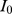 denotes the incident x-ray intensity, 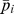 is the mean of the sinogram data at bin 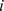 and 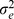 is the background electronic noise variance.

### Overview of the present ndiTV prior

Inspired by the PICCS algorithm introduced by Chen *et al* [15], in this paper, we propose a ndiTV prior by incorporating the ndiNLM filter proposed by Ma *et al* [38], which is expressed as follows:
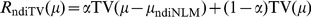
(3)where 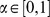 is a scalar factor and 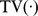 denotes the total variation operator and is defined as follows:
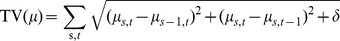
(4)where 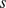 and 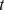 are the indices of the location of the attenuation coefficients of the desired-image. 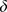 is a small constant used for keeping differentiable with respect to image intensity. The term 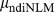 in equation (3) represents the ndiNLM filter and can be written as follows:
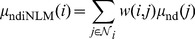
(5)where 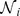 denotes the search-window and 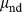 denotes the normal-dose prior image. The weight 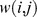 quantifies the similarity between pixel 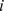 in the image domain 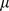 and pixel 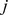 in the prior image domain 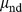, respectively, and can be expressed as follows:
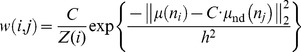
(6)

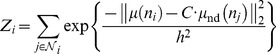
(7)where 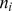 and 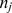 denote two local similarity neighborhoods (named patch-windows) centered at pixels 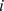 and 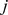, respectively. The terms 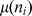 and 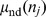 denote the vectors of neighborhood pixel values restricted in the patch-windows 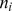 and 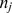, respectively. The notation 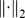 denotes a Euclidean distance between two similarity patch-windows. The parameter 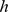 is a factor controlling the decay of the exponential function.

In equation (6), 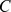 is a local compensation factor used accounting for local intensity change between the desired- and prior- images, *i.e.*,
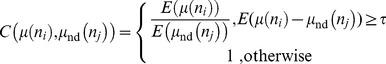
(8)where 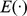 denotes the expected value or mean of the intensity in the patch-window 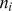, and the threshold factor 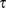 is determined by estimating the standard deviation of homogeneous area near the patch-window neighborhood of the current image estimation.

In summary, the objective function of the present PWLS-ndiTV approach can be written as follows:
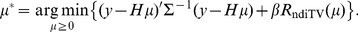
(9)


### Implementation of the PWLS-ndiTV approach

Due to the nonlinear form of the ndiNLM filter with respect to image intensity, general optimization algorithm is difficult to effectively minimize the objective function in equation (9). To solve this problem, in this paper, similar to our previous works [13], [43], an alternating minimization scheme was used to optimize (9) wherein the weights 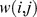 in equation (5) can be automatically updated according to the similarity between the patch-windows in the current estimation 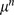 (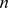 is the iterative index) and the normal-dose prior image 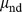 during each iteration. In summary, the present PWLS-ndiTV approach for CT image reconstruction has three main steps as follows:

**1) Prior estimation.** Given a current estimation 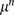, 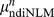 is calculated by performing the ndiNLM filter on the current estimation 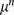 using the prior image 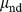.

**2) Steepest descent optimization.** For minimizing the objective function of the PWLS-ndiTV, a steepest descent optimization algorithm is used to yield new image estimation, *i.e.*, 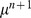, which is described as follows:
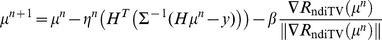
(10)where 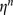 represents the gradient step-size. 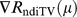 represents the gradient of 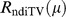 and 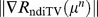 is the relative normalization factor.

**3) Cycle Update.** Update 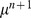 using the aforementioned step in each cycle until stop criteria is satisfied.

In the implementation, the scalar factor 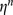 was calculated adaptively by using the following estimator [44]:
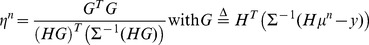
(11)



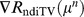
 in (10) is calculated as follows:
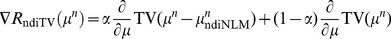
(12)


where 
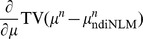
 and 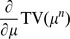 in equation (12) can be calculated using the following gradient operator with a small positive scalar 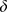:
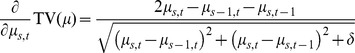


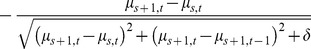


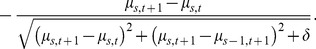
(13)


In addition, the preliminary image reconstructed by the FBP method with ramp filter was used as the initial estimation for all iterative algorithms. The threshold factor 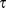 in equation (8) was selected by using the current image estimation of each update. The total number of iteration 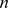 was set as 100 for yielding stable image estimation.

### Data acquisitions

To validate and evaluate the performance of the PWLS-ndiTV for CT image reconstruction from sparse-view CT measurement, a modified Shepp-Logan phantom digital NURBS-based cardiac-torso (NCAT) phantom [45] and an anthropomorphic torso phantom were used for experimental data simulations.

#### Modified Shepp-Logan phantom

**Figure 1:**
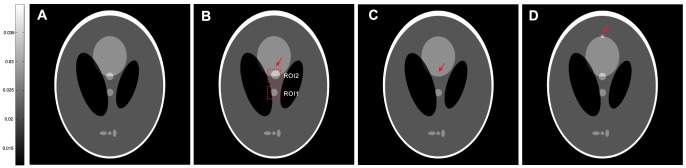
Four modified Shepp-Logan phantoms used in the studies. (A) is the standard modified phantom; (B) is the second modified phantom wherein motion is designed compared to (A); (C) is the third modified phantom wherein an object is removed compared to (A); and (D) is the fourth modified phantom with a low-contrast lesion added compared to (A). All the images are displayed in a same window [0.0122, 0.0398].

Fig. 1 shows four modified 2D Shepp-Logan phantoms. Fig. 1A shows the standard modified phantom. Fig. 1B shows the second modified phantom wherein a motion object as indicated by the arrow is included compared to Fig. 1A. Fig. 1C shows the third modified phantom wherein an object is removed compared to Fig. 1A as indicated by the arrow and Fig. 1D shows the fourth modified phantom used for the receiver operating characteristic (ROC) study, which contains a low-contrast small lesion as indicated by the arrow compared to Fig. 1A. The density of lesion with a radius of 3.0 mm is 1.5% above the background density. Each phantom is composed by 512 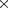 512 square pixels with each pixel size of 1.25 mm 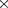 1.25 mm.

#### Digital NCAT phantom

**Figure 2:**
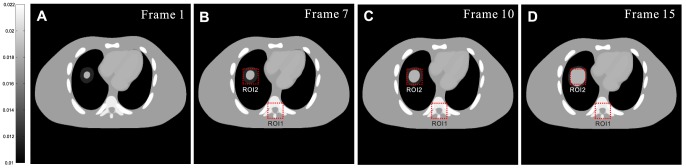
Digital NCAT phantom images at four different frames. The images (A)-(D) correspond to the frame 1, 7, 10 and 15, respectively. All images are displayed in a same window [0.01, 0.022].

Fig. 2 shows four frames of the dynamic NCAT phantom used in our study. Due to respiratory motion and cardiac motion, each frame is different. Fig. 2A shows the first frame of sequential CT images, which is used for simulating the normal-dose prior image for image reconstruction of other frames as shown in Fig. 2B-D. Significant motion deformation can be observed between other three frames and the first one. Each phantom is composed by 512 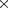 512 square pixels with each pixel size of 0.6 mm 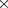 0.6 mm.

#### Data acquisition by simulation

Without loss of generality, we chose a geometry that was representative for a mono-energetic fan-beam CT scanner setup with a circular orbit to acquire 1,160 views over 2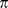. The number of channels per view was 672. The distance from the rotation center to the x-ray source is 570 mm and the distance from the X-ray source to the detector is 1,040 mm. Each projection datum along an x-ray through the sectional image is computed based on the known densities and intersection areas of the ray with the geometric shapes of the objects in the sectional image. For the noisy projection data, similar to the study [13], [46], after calculating the noise-free line integral 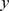 as a direct projection operation, the noisy measurement 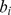 at each bin 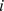 was generated according to the following statistical model of pre-logarithm projection data
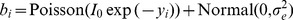
(14)where 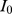 denotes the incident x-ray intensity and 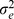 is the background electronic noise variance. In this study, for two phantoms, the x-ray exposure level 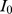 was all set to 9.0
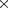
10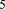 and 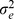 was all set to 10 for normal-dose scan simulation. The noisy measurement 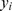 was calculated by performing the logarithm transform on 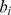. For the Shepp-Logan phantom and NCAT phantom experiments, the sparse-view projections were generated by under-sampling the 1,160 views of normal-dose simulation to only 25 views evenly over 2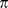.

#### Anthropomorphic torso phantom

**Figure 3:**
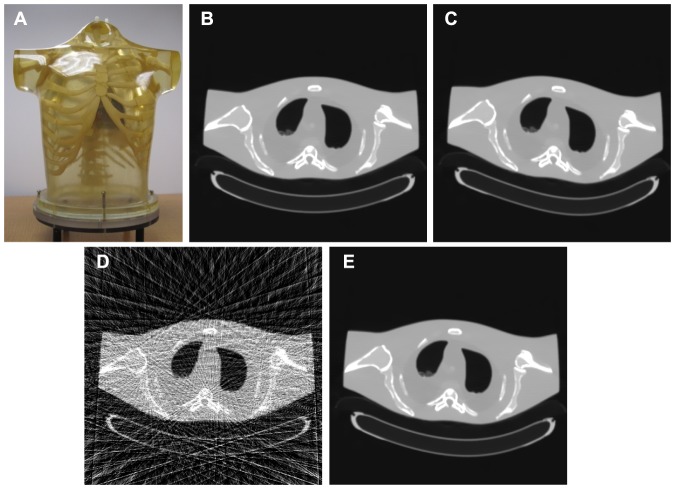
An anthropomorphic torso phantom used in the studies. (A) is the physical phantom illustration; (B) is the image reconstructed by a FBP apporach with ramp filter from the full 1160-views projection data; (C) is a deformed image from the image (B), which is used as the prior image for sparse-view CT image reconstruction with the PWLS-PICCS and PWLS-ndiTV approaches; (D) is the image reconstructed by the FBP apporach with ramp filter from the 58-views projection data; and (E) is the registered image between (C) and (D) which is used for sparse-view CT image reconstruction with the PWLS-RPICCS approach.

An anthropomorphic torso phantom (Radiology Support Devices, Inc., Long Beach, CA) as shown in Fig. 3A was used for experimental data acquisition. The phantom was scanned by a clinical CT scanner (Siemens SOMATOM Sensation 16 CT) in a cine mode at a fixed bed position with a protocol of 100 mAs and 120 kVp. The associated imaging parameters of the CT scanner were as follows: (1) each rotation included 1,160 projection views evenly spaced on a circular orbit; (2) each view contained 672 data elements each from one of the 672 detector bins; (3) the distance from the detector arrays to the X-ray source was 1,040 mm; (4) the distance from the rotation center to the X-ray source was 570 mm; and (5) the space of each detector bin was 1.407 mm. In this study, the sparse-view projections were generated by under-sampling the 1,160 views to only 58 views evenly over 2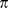. Fig. 3B shows a CT image reconstructed by a FBP method with ramp filter from the full 1160-views projection data as a gold-standard reference. Fig. 3C shows an elastic deformed CT image from Fig. 3B, which is used as the prior image for sparse-view CT image reconstruction with the PWLS-PICCS and the PWLS-ndiTV approaches. Fig. 3D shows a CT image reconstructed by the FBP method with ramp filter from the 58-views projection data. Fig. 3E shows the registered image between the original prior image (*i.e.*, Fig. 3C) and the FBP image (*i.e.*, Fig. 3D) by using the B-spline based image registration technique [47], which is used as the prior image for sparse-view CT image reconstruction with the PWLS-RPICCS approach.

### Performance evaluation

#### Evaluation by noise reduction

The following three metrics were utilized to evaluate the noise reduction: (1) peak signal-to-noise ratio (PSNR); (2) mean per cent squared error (MPSE); and (3) mean per cent absolute error (MPAE):
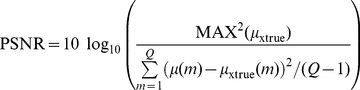
(15)

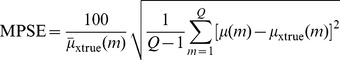
(16)

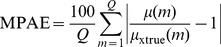
(17)where 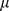 denotes the to-be-reconstructed image, 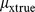 denotes the ground truth image, 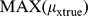 represents the associated maximum intensity value, and 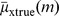 denotes the associated average pixel value in the interest of region (ROI) wherein 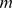 indexes the pixels in the ROI. 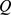 is the number of pixels in the ROI.

#### Evaluation by reconstruction accuracy

The rRMSE (relative root mean square error) measurements of the reconstructions were carried out to quantify the accuracy of the reconstruction. The rRMSE is defined as:
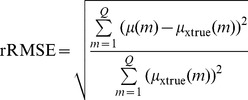
(18)where 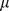 denotes the to-be-reconstructed image, 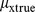 denotes the ground truth image. 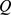 is the number of pixels in the ROI.

#### Resolution-noise tradeoffs

The image resolution is analyzed by the edge spread function (ESF). Using the strategy described in [48] and under the assumption that the broadening kernel is a Gaussian function with standard deviation 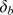, an error function (erf) is used to represent the ESF function parameterized by 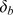. The parameter 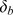 is calculated by fitting the vertical profiles to an erf function, and then the associated full-width at half-maximum (FWHM) of the Gaussian broadening kernel is denoted as 2.35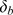 which is used to represent the to-be-reconstructed image resolution. The noise-resolution tradeoff curves were generated from the simulated projection data using the modified phantom. In addition, the noise level of the to-be-reconstructed image was characterized by the standard deviation of a uniform region of size 20
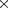
20 in the background region. By varying the penalty parameter 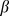 settings, we obtained the associative noise-resolution tradeoff curves from the reconstructed images.

#### Receiver operating characteristic study

The ability of lesion detection is a general principle for evaluating a medical imaging system. Extensive experimental results have demonstrated that a ROC curve can provide a comprehensive and useful description by exploring the combinations of sensitivity and specificity in a diagnostic test. In practice, after generating a variety of pairs of true positive fraction (TPF) and false positive fraction (FPF), the ROC curve can be drawn or fitted from the obtained TPF and FPF values [49]. Then, the total area under each curve is calculated. The associated index is often named as “AUC” and larger AUC usually reflects better lesion detection. To eliminate the intra human observer variation, a channelized Hotelling observer (CHO) can be employed to generate the ROC curves [50] and the series of ratings from the output can be subsequently analyzed by using the ROCKIT package with bi-normal model (http://metz-roc.uchicago.edu/). In this paper, ROC studies are performed from the computer-generated data by adding a low-contrast small lesion in a modified Shepp-Logan phantom as indicated by an arrow in Fig. 1D. To evaluate the ability of lesion detection, a total of 100 normal-dose projection data with full views were generated according to equation (14) using the modified Shepp-Logan phantom with and without the low-contrast region. The associated images were reconstructed by different approaches from the same sparse-view (*i.e.*, 25-views) projection data by under-sampling 1,160 views, respectively.

### Comparison approach and parameter settings

To validate and evaluate the performance of the present PWLS-ndiTV approach, the PICCS approach described in [15] was also carried out under the PWLS criteria for comparison, which is referred to as “PWLS-PICCS”. In addition, the PWLS-PICCS approach combining a registration step were also carried out for comparison, which is referred to as “PWLS-RPICCS”. By incorporating the noise model described in equation (2), the objective function of the PWLS-PICCS can be written as follows:
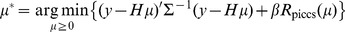
(19)where 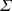 is a diagonal matrix with the 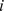
th element of 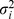 which is estimated in equation (2). 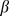 is a hyper-parameter. 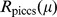 denotes a PICCS prior term and is defined as follows:
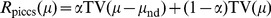
(20)where the term 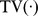 denotes the total variation operator which is defined in equation (4) and 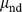 denotes the prior image. 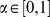 is the relative weight of two terms. Comparing with the PWLS-PICCS approach, the PWLS-RPICCS approach uses the B-spline based image registration technique [47] as a preprocessing step to minimize the misalignment between the prior- and desired- images.

The related parameters in the implementation were set as follows: (1) for the PWLS-ndiTV approach, the size of “patch-window” (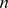) was 5 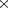 5, the size of “search-window” (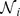) was 23 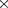 23, the parameter 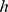 was set manually with noise reduction measure; (2) for the PWLS-ndiTV, PWLS-PICCS and PWLS-RPICCS approaches, the hyper-parameter 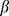 was selected manually with noise reduction measure, and the relative weight 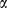 was 0.5 in almost experiments except in subsection for discussing the influence of 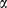 on the reconstruction accuracy.

All the algorithms were implemented in Matlab 7.14 (The Math Works, Inc.) programming environment. The codes were run on a typical desktop computer with Intel Pentium G620 Processor, 2.60 GHz and 2 GB of RAM memory.

## Results

### Modified Shepp-Logan phantom studies

#### Visual inspection

**Figure 4:**
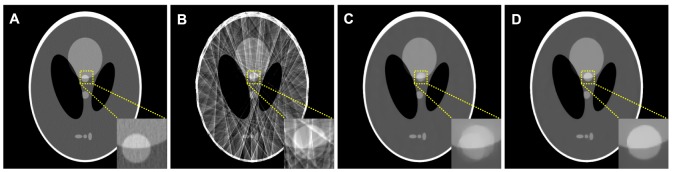
Shepp-Logan phantom reconstructions by different methods. (A) is the image reconstructed by the FBP approach with ramp filter from the full noise-free projection data, which is used as the prior image; (B) is the image reconstructed by the FBP approach with ramp filter from the 25-views projection data; (C) is the image reconstructed by the PWLS-PICCS approach (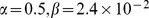) from the 25-views projection data; and (D) is the image reconstructed by The PWLS-ndiTV approach (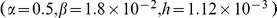) from the 25-views projection data. All the images are displayed in a same window [0.0122, 0.0398].

**Figure 5:**
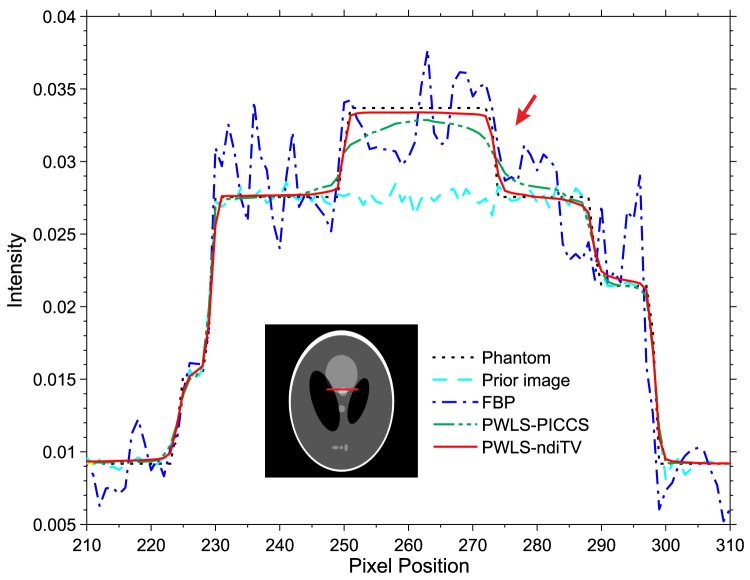
The profiles located at the pixel positions x from 200 to 310 and y = 410. The “dot-dashed line” denotes the profile from the FBP approach; the “dot-dot-dashed line” denotes the profile from the PWLS-PICCS approach; the “solid line” denotes the profile from the PWLS-ndiTV approach; the “dotted line” denotes the profile from the ground truth; and the “dashed line” denotes the profile from the normal-dose prior image.

Fig. 4A shows the image reconstructed by the FBP approach with ramp filter from the simulated full normal-dose projection data, which is used as the prior image. A noticeable difference between the normal-dose prior image (*i.e.*, Fig. 4A) and the desired-image (*i.e.*, Fig. 1B) can be observed. Fig. 4B shows the image reconstructed by the FBP approach with ramp filter from the 25-views projection data. Serious streak artifacts can be observed due to the sparse-view projection data measurements. Fig. 4C and D show the images reconstructed by the PWLS-PICCS (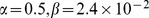) and the PWLS-ndiTV (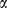 = 0.5, 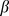 = 1.8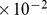, 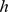 = 1.12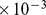) approaches, respectively. To further demonstrate the performance of the PWLS-PICCS and PWLS-ndiTV approaches, a ROI was zoomed and displayed in the bottom right corner of each figure. It can be clearly seen that the PWLS-ndiTV achieves remarkable gains than the PWLS-PICCS in terms of maintaining the structure information of ROI. In other words, the PWLS-ndiTV can reduce the influence of the misalignment from the prior image as comparison with the PWLS-PICCS. Furthermore, Fig. 5 displays the profiles from different approaches. It can be observed that the profile from the PWLS-ndiTV matches well with that from the ground truth. The results indicate that the gains from the PWLS-ndiTV are more noticeable compared with those from the PWLS-PICCS.

#### Noise reduction measure

Table 1 lists the PSNR, MPSE, and MPAE measures of the images (as shown in Fig. 4) reconstructed by the FBP, PWLS-PICCS and PWLS-ndiTV approaches from the 25-views projection data. Two ROIs, as indicated by two squares in Fig. 1B, represent the matched and mismatched regions between the desired- and prior- image, respectively. In the matched region (ROI1), the results from both the PWLS-PICCS and PWLS-ndiTV approaches exhibited similar results with more than 50% gains over that from the FBP approach in terms of the PSNR, MPSE, and MPAE measures. Meanwhile, in the mismatched region (ROI2), the PWLS-ndiTV outperformed the PWLS-PICCS with more than 20% gains in terms of the MPSE and MPAE measures and with more than 10% gains in terms of the PSNR measure.

**Table 1:** Image quality metrics on two ROIs as indicated by the squares in Fig. 1B.

Methods	Matched regions (ROI1)			Mismatched regions (ROI2)		
	PSNR	MPSE	MPAE	PSNR	MPSE	MPAE
FBP	17.54	5.21	3.48	16.78	6.77	4.54
PWLS-PICCS	29.14	0.36	0.24	23.57	0.67	0.75
PWLS-ndiTV	28.64	0.39	0.26	26.54	0.48	0.56

#### Influence of misalignments on the reconstruction accuracy

**Figure 6:**
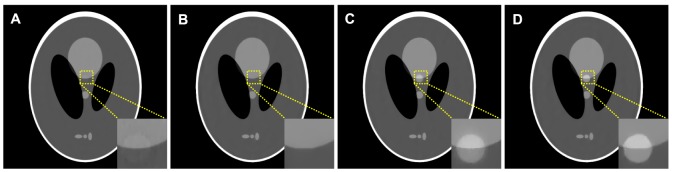
Shepp-Logan phantom reconstructions by different methods from the 25-views projection data. (A) and (B) are the images reconstructed of the case one by the PWLS-PICCS (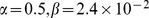) and PWLS-ndiTV (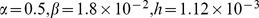) approaches from the 25-views projection data, respectively; (C) and (D) are the images reconstructed of the case two by the PWLS-PICCS (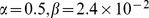) and PWLS-ndiTV (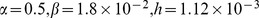) approaches from the 25-views projection data, respectively. All the images are displayed in a same window [0.0122, 0.0398].

To demonstrate the influence of misalignments between the desired- and prior- image on the reconstruction accuracy from the PWLS-PICCS and PWLS-ndiTV approaches, we simulated two extreme cases by modifying the Shepp-Logan phantom as shown in Fig. 1A and C. For the case one, Fig. 1A was used to simulate the prior image for reconstructing the desired-image of Fig. 1C. Meanwhile, for the case two, Fig. 1C was used to simulate the prior image for reconstructing the desired-image of Fig. 1A. Fig. 6 shows the corresponding results reconstructed by the PWLS-PICCS and PWLS-ndiTV approaches from the 25-views projection data. The results of case one are shown in Fig. 6A and B, and the results of case two in Fig. 6C and D. We can see that the reconstruction accuracy is significantly influenced by the misalignments between the desired- and prior- image in terms of visual inspection appealing compared with two ideal phantoms. However, the PWLS-ndiTV can yield remarkable gains over the PWLS-PICCS in terms of the edge-preserving ability around the mismatched regions.

#### Influence of the parameter 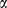 on the reconstruction accuracy

**Figure 7:**
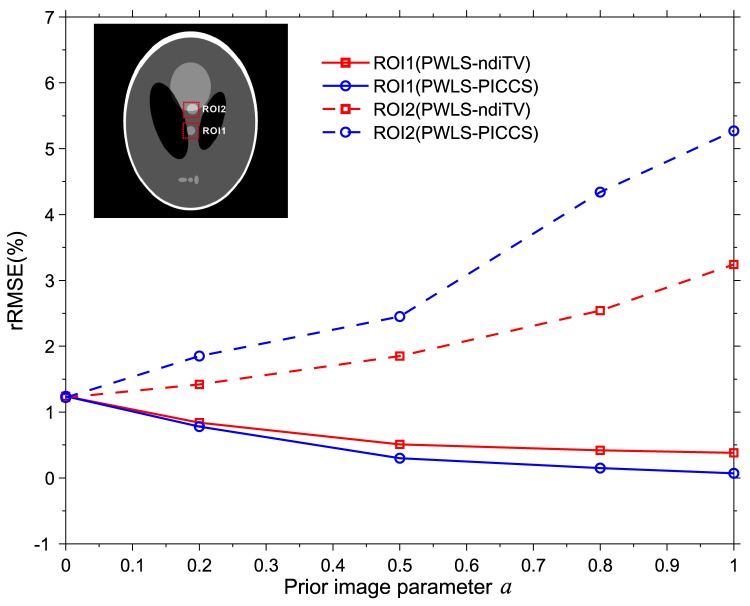
The rRMSE measures within two ROIs. ROI1 is the matched region and ROI2 is the mismatched region.

For the PWLS-ndiTV and PWLS-PICCS approaches, the influence of the parameter 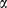 on the reconstruction accuracy should be considered carefully because the performance of two approaches heavily depends on the 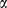 setting. In this study, two approaches were validated quantitatively from the same 25-view projection data with a range of 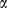 (*i.e.*, 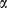 = 0, 0.3, 0.5, 0.8, 1) at different 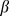 setting. Fig. 7 shows the rRMSE measurements of two ROIs as indicated by squares in Fig. 1B. It can be observed that in the matched region (ROI1), the rRMSEs from two approaches are decreased as 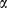 increasing and the PWLS-PICCS outperforms PWLS-ndiTV slightly. The results demonstrate that the PWLS-PICCS can yield slight gains over the PWLS-ndiTV in the matched region reconstruction. However, in the mismatched region (ROI2), the PWLS-ndiTV can achieve significant gains over the PWLS-PICCS with remarkable deviation suppression. In practice, there exists a tradeoff between the reconstruction accuracy of matched and mismatched regions [50]. In our present experiments, we found 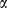 = 0.5 was proper.

#### Noise-resolution tradeoff

**Figure 8:**
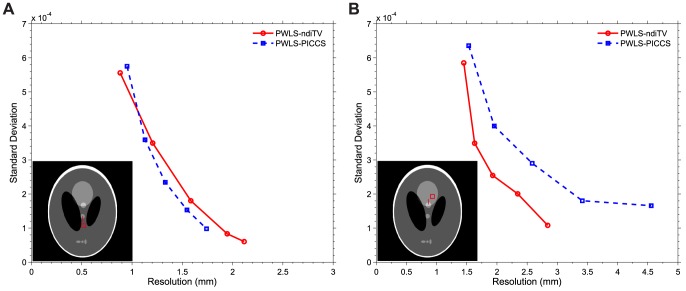
Noise-resolution curves of the PWLS-PICCS and PWLS-ndiTV approaches.

Fig. 8 shows the noise-resolution curves of the PWLS-PICCS and PWLS-ndiTV approaches. Two different vertical profiles as indicated by two lines in the images located at the left bottom of Fig. 8A and B were selected to represent the matched and mismatched regions between the desired- and prior- image, respectively. Additionally, two uniform regions near the corresponding profiles as indicated by squares in the background were selected for calculating the standard deviation of the reconstructed image. It can be seen that for the matched ROI in Fig. 8A, the PWLS-ndiTV and PWLS-PICCS approaches achieved similar changing tendency of the noise-resolution curves. Meanwhile, the PWLS-ndiTV yielded noticeable gains over the PWLS-PICCS for the mismatched ROI in Fig. 8B in terms of the noise-resolution tradeoff curve. In this study, the parameter 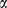 was fixed at 0.5 for the PWLS-ndiTV and PWLS-PICCS approaches, the hyper-parameter 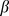 for the PWLS-PICCS approach was set from  to 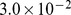 and the hyper-parameter 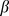 for the PWLS-ndiTV approach was set from 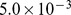 to 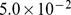.

#### ROC curve

**Figure 9:**
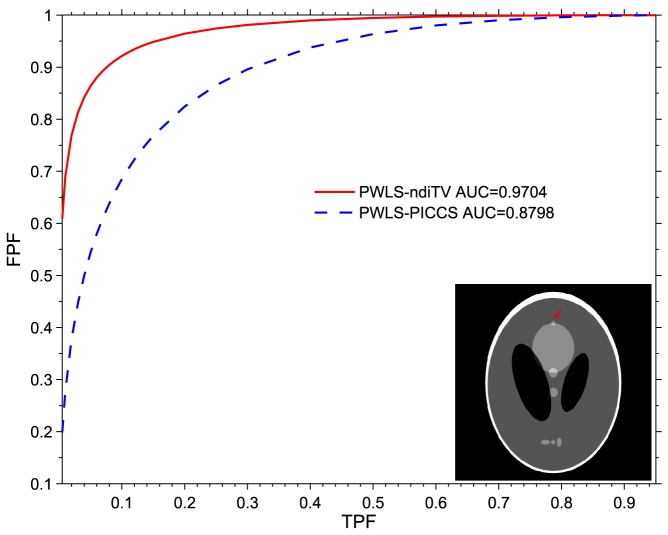
ROC curves of the PWLS-PICCS and PWLS-ndiTV approaches.

Fig. 9 shows the ROC curves from the PWLS-PICCS and PWLS-ndiTV approaches. The area under the ROC curve from the PWLS-ndiTV is 0.9704 whereas the area under the ROC curve from the PWLS-PICCS is 0.8798. The results indicate that the PWLS-ndiTV slightly outperformed PWLS-PICCS in terms of detectability of abnormality in low-contrast diagnosis. In this study, 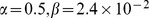 were set for the PWLS-PICCS approach and 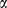 = 0.5, 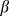 = 1.8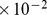,  = 1.12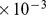 were set for the PWLS-ndiTV approach.

### Dynamic NCAT phantom studies

#### Visual inspection

**Figure 10:**
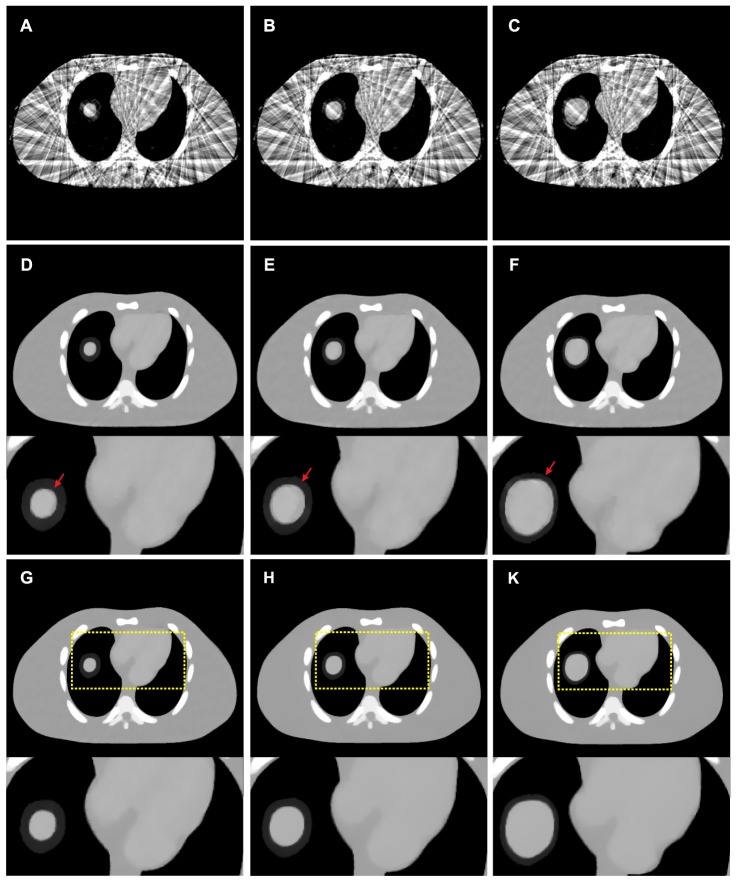
Dynamic NCAT phantom reconstructions by different approaches from the 25-views projection data. (A)–(C) are the images of frame 7, 10 and 15 reconstructed by the FBP approach with ramp filter; (D)–(F) are the images of frame 7, 10 and 15 reconstructed by the PWLS-PICCS approach (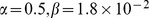); and (G)–(K) are the images of frame 7, 10 and 15 reconstructed by the PWLS-ndiTV approach (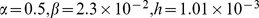). All the images are displayed in a same window [0.01, 0.022].

**Figure 11:**
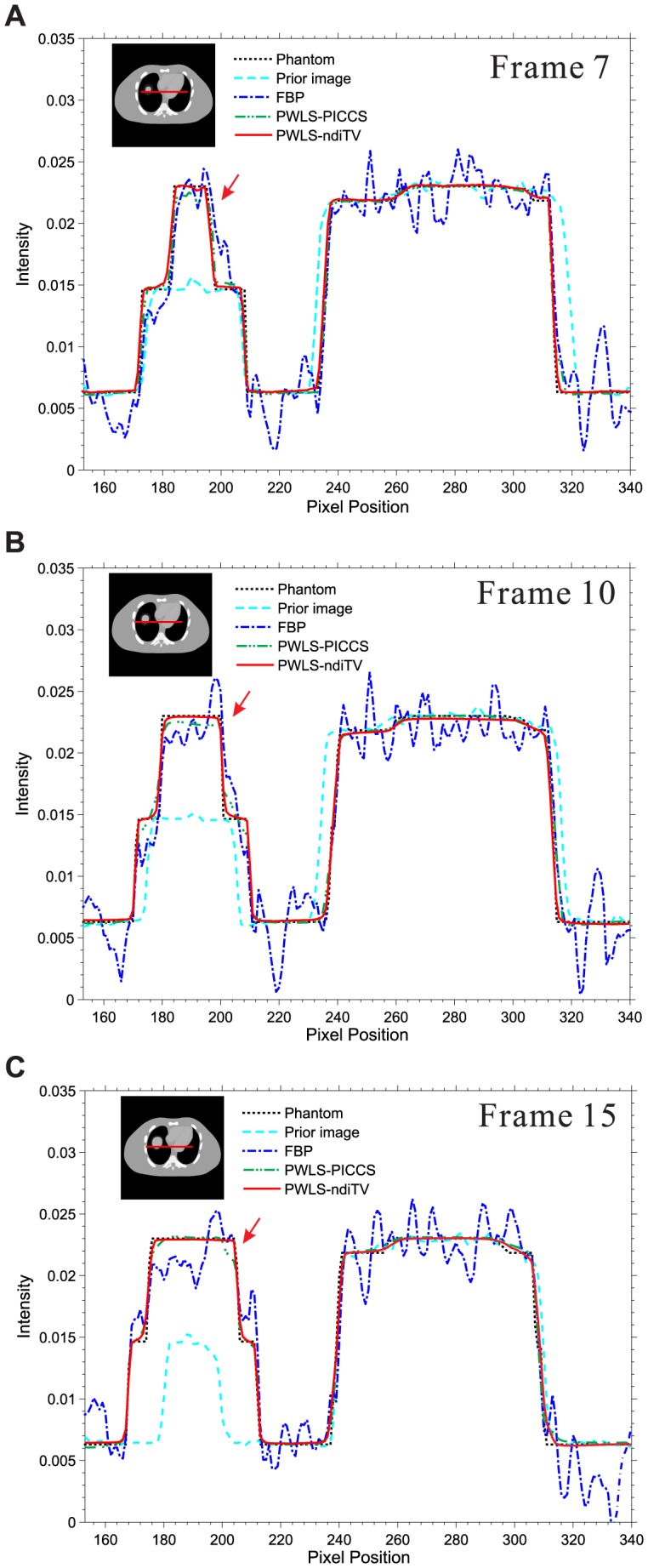
Profiles from different results at different frames in Fig. 10. (A) is the profiles from frame 7 located at the pixel positions 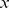 from 153 to 340 and 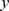 = 247; (B) is the profiles from frame 10 located at the pixel positions 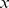 from 153 to 340 and 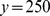; and (C) is the profiles from frame 15 located at the pixel positions 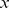 from 153 to 340 and 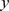 = 257. The “dot-dashed line” denotes the profile from the FBP approach; the “solid line” denotes the profile from the PWLS-PICCS or PWLS-ndiTV approaches; the “dotted line” denotes the profile from the ground truth; and the “dashed line” denotes the profile from the normal-dose prior image.

Fig. 10A-C show the images of frames 7, 10 and 15 reconstructed by the FBP approach with ramp filter from the 25-views projection data, respectively. Serious artifacts can also be observed similar to the results from the study of Shepp-Logan phantoms. Fig. 10D-F show the images of frames 7, 10 and 15 reconstructed by the PWLS-PICCS approach (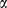 = 0.5, 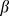 = 1.8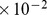) from the 25-views projection data, respectively. Consequently, Fig. 10G-K show results reconstructed by the PWLS-ndiTV approach (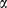 = 0.5, 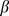 = 2.3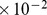,  = 1.01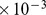) from the 25-views projection data. The zoomed ROIs as indicated by the squares are included in Fig. 10. It can be seen that the edges of vessels and cardiac from the PWLS-PICCS existed a noticeable deviation from the desired ones as comparison with that from the PWLS-ndiTV. This phenomenon is more obvious in the regions as indicated by arrows in Fig. 10 where the misalignments exist. Fig. 11 displays the profiles from different results at different frames. It can be observed that the profiles from the PWLS-ndiTV match better with that from the PWLS-PICCS. In other words, the PWLS-ndiTV can yield more gains over the PWLS-PICCS in terms of the edge details preserving.

#### Noise reduction measure

Table 2 lists the PSNR measures of the images as shown in Fig. 10 reconstructed by the FBP, PWLS-PICCS, and PWLS-ndiTV approaches from the 25-views projection data. Two ROIs, as indicated by two squares in Fig. 2B–D, represent the matched and mismatched regions, respectively. It can be seen that, for the three frames, the results from both the PWLS-PICCS and PWLS-ndiTV approaches exhibited similar results of more than 50% gains over that from the FBP approach in the matched region (ROI1). And in the mismatched region (ROI2), the PWLS-ndiTV approach outperformed the PWLS-PICCS approach with more than 10% gains.

**Table 2:** PSNR measures on two ROIs as indicated by the squares in Fig. 2B–D.

Methods	Matched regions (ROI1)			Mismatched regions (ROI2)		
	Frame 7	Frame 10	Frame 15	Frame 7	Frame 10	Frame 15
FBP	11.93	11.45	12.13	10.48	10.73	11.01
PWLS-PICCS	25.64	25.45	25.52	21.56	21.14	20.95
PWLS-ndiTV	25.21	24.78	24.93	23.88	23.54	23.78

#### Comparison studies with the PWLS-RPICCS approach

**Figure 12:**
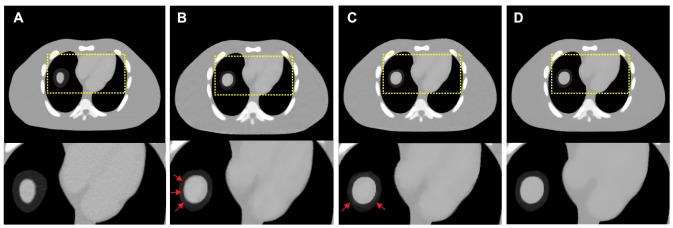
Comparison studies with the PWLS-RPICCS approach. (A) is the registered image between frame 10 and 1, which is used as the prior image for the PWLS-RPICCS approach; (B) is the frame 10 reconstructed by the PWLS-PICCS approach (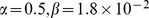) using Fig. 2A as the prior image from the 25-views projection data; (C) is the frame 10 reconstructed by the PWLS-RPICCS approach (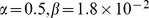) using Fig. 12A as the prior image from the 25-views projection data; and (D) is the frame 10 reconstructed by the PWLS-ndiTV approach (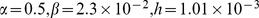) using Fig. 2A as the prior image from the 25-views projection data. All the images are displayed in a same window [0.01, 0.022].

**Figure 13:**
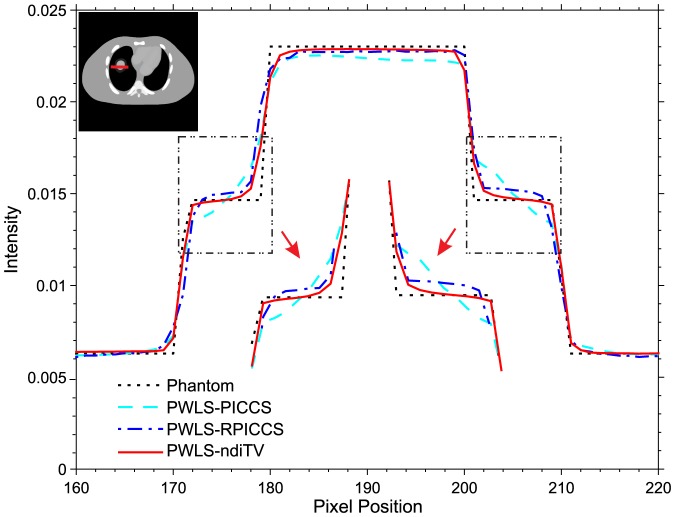
The profiles located at the pixel positions x from 160 to 220 and y = 250. The “dotted line” denotes the profile from the ground truth; the “dashed line” denotes the profile from the PWLS-PICCS approach; the “dot-dashed line” denotes the profile from the PWLS-RPICCS approach; and the “solid line” denotes the profile from the PWLS-ndiTV approach.

In this section, the comparison studies between the PWLS-ndiTV and PWLS-RPICCS approaches were performed on the NCAT phantom. The frame 10 (*i.e.*, Fig. 10B) was used as the objective image and the frame 1 (*i.e.*, Fig. 2A) was used as the prior image. The misalignment between the frames 10 and 1 was reduced by the B-spline based image registration technique [47]. Fig. 12A shows the registered image between frame 10 and 1, which is used as the prior image for the PWLS-RPICCS approach. Fig. 12B shows the frame 10 reconstructed by the PWLS-PICCS approach using Fig. 2A as the prior image from the 25-views projection data. Fig. 12C shows the frame 10 reconstructed by the PWLS-RPICCS approach using Fig. 12A as the prior image from the 25-views projection data. Fig. 12D shows the frame 10 reconstructed by the PWLS-ndiTV approach using Fig. 2A as the prior image from the 25-views projection data. Furthermore, the profiles shown in Fig. 13 illustrate that the present PWLS-ndiTV achieves more noticeable gains than both the PWLS-PICCS and PWLS-RPICCS in preserving the edge details as indicted by the arrows. To quantitatively evaluate above three approaches, Table 3 lists the image quality metrics of two ROIs, where the ROI1 and ROI2 represent the matched and mismatched regions as shown in Fig. 2C, derived from the sparse-view CT image reconstruction by three different methods. For the matched region (ROI1), the gains from the PWLS-PICCS and PWLS-RPICCS are similar and slight over that from the present PWLS-ndiTV in terms of three image quality metrics. Meanwhile, for the mismatched region (ROI2), the present PWLS-ndiTV can achieve noticeable gains than the other two approaches in terms of three image quality metrics. The results have demonstrated that for the PWLS-RPICCS approach, the gains from the registration technique in the mismatched regions are limited in improving image reconstruction performance as compared with the present PWLS-ndiTV approach.

**Table 3:** Image quality metrics on two ROIs as indicated by the squares in Fig. 2C.

Methods	Matched regions (ROI1)			Mismatched regions (ROI2)		
	PSNR	MPSE	MPAE	PSNR	MPSE	MPAE
PWLS-PICCS	25.49	0.34	0.28	21.14	0.51	0.69
PWLS-RPICCS	25.45	0.35	0.26	22.67	0.46	0.65
PWLS-ndiTV	24.52	0.40	0.31	23.54	0.43	0.59

### Anthropomorphic torso phantom studies

**Figure 14:**
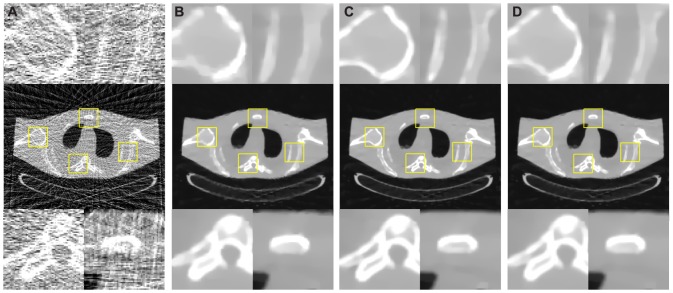
Anthropomorphic torso phantom reconstructions by different approaches from the 58-views projection data. (A) is the image reconstructed by the FBP approach with ramp filter; (B) is the images reconstructed by the PWLS-PICCS approach (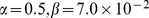) using Fig. 3C as the prior image; (C) is the images reconstructed by the PWLS-RPICCS approach (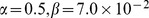) using Fig. 3E as the prior image; and (D) is the image reconstructed by the PWLS-ndiTV approach (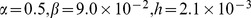) using Fig. 3C as the prior image. All the images are displayed in the same window [0, 0.024].

Fig. 14 shows the results reconstructed by different approaches from the 58-views projection data. Fig. 14A shows the image reconstructed by the FBP approach with ramp filter. Serious streak artifacts can be observed. Fig. 14B shows the images reconstructed by the PWLS-PICCS approach using Fig. 3C as the prior image. Fig. 14C shows the images reconstructed by the PWLS-RPICCS approach using Fig. 3E as the prior image. Fig. 14D shows the image reconstructed by the PWLS-ndiTV approach using Fig 3C as the prior image. To further evaluate the performance of different approaches, four regions of interest indicated by the squares were zoomed and displayed in Fig. 14. It can be seen that the PWLS-PICCS approach yielded the result with noticeable blurred effects in the mismatch regions between the reconstructed and prior images. However, the PWLS-ndiTV and the PWLS-RPICCS approaches can achieve similar gains in term of the edges information preservation.

## Discussion

Statistical iterative reconstruction (SIR) for x-ray CT has been extensively explored for radiation dose reduction in CT field [10], [27], [42], [51], [52], [53]. Usually, the objective function of SIR with unconditional constrains has two terms: one is “data-fidelity term”, which is developed by incorporating the statistical measurement model, and another is “prior term” or “penalty term”, which is commonly designed by considering the properties of the desired-image itself. For sparse-view CT image reconstruction, the data enforcement step and the minimization step are often implemented in an alternating manner using the POCS or SART algorithm [15], [16]. The major drawback of the POCS or SART algorithm is that the statistical properties of CT measurement cannot be well considered in the implementation. To express this problem, Tang *et al* [17] has proved that the PWLS approach with a TV-based prior term outperforms the conventional PWLS approach with quadratic prior term from the sparse-view measurements in terms of streak artifacts suppression. Lauzier and Chen [54], [55] also demonstrated that the PICCS strategy with accurate projection data noise modelling can yield better performance on restoring spatial resolution in time-resolved contrast enhanced CT image reconstruction and obtaining more uniform noise spatial distribution in low-dose image reconstruction.

High-quality CT measurements for a patient acquired in previous scans can be utilized as prior knowledge to facilitate the subsequent image reconstruction, for instance, in the cases of low-dose scan and sparse-view measurements [13], [15], [26], [27], [28], [31], [33], [35], [38]. However, due to the inverse effect of the mismatched regions between the desired- and prior- image, using prior image without any misalignment reduction would lead to blur or even loss of the original structures in mismatched regions [33]. In this paper, we propose an improved version of PICCS strategy by incorporating the ndiNLM filter based on the patch-based search mechanism for dealing with the inverse effect of mismatched area. Experimental results have demonstrated that the PWLS-ndiTV approach can preserve the detailed structure of the desired-image in the mismatched regions.

For the developed PWLS-ndiTV algorithm, we would like to make the following discussions. First, the PWLS-ndiTV algorithm is a version of the widely used one-step-late (OSL) iteration algorithm, and it is similar to our previous work [13], [43], the binary optimal reconstruction strategy was used for solving the objective function. Just like many existing OSL algorithms, the present algorithm also lacks strict global convergence proof. But, it is worth to note that extensive experiments suggest that the present algorithm is still effective for searching at least a local minimum in practice. Second, due to the introduction of ndiNLM filter, several scalar parameters should be carefully tuned in the present PWLS-ndiTV algorithm. For example, to reduce the computational load, the search-window should be limited to an appropriate non-local neighborhood system, and to avoid over-smooth of image, the controlling parameter 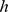 can be not so large. In our present study, by extensive experiments with visual inspection and quantitative measurements, we found that a 23
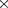
23 search-window and a 5
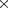
5 patch-window are adequate for effective noise and artifacts suppression while retaining computational efficiency.

Third, the drawback of the present PWLS-ndiTV approach is its computational burden due to the ndiNLM filter compared with the PWLS-PICCS approach. For example, in the case of 2D Shepp-Logan image reconstruction from the 25-views projection, the PWLS-ndiTV approach with a 23
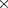
23 search-window and a 5
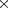
5 patch-window takes about 0.5 min to finish one iteration to reconstruct the image of size 512
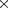
512 using a PC with 2.60 GHz CPU. Meanwhile, the PWLS-RPICCS approach takes about 15 min to perform the registration operation before performing image reconstruction. The results show that the PWLS-ndiTV takes acceptable computational cost as comparison with the PWLS-RPICCS for sparse-view CT image iterative reconstruction. Additionally, several techniques proposed by Coupe *et al* [56] can be used for optimizing the ndiNLM algorithm including block-wise implementation and parallel computation, and the graphic processing unit (GPU) implementation would also be a sound solution for solving this problem. Last, the experiments were performed on the 2D digital phantoms and anthropomorphic torso phantom. Practically, the 3D/4D phantoms and in vivo data need to be studied, which would be an interesting topic for future research.

## Conclusions

In this paper, we present a PWLS-ndiTV approach for sparse-view CT image reconstruction in the case of a known normal-dose image. The aim of the present approach is to relieve the requirement of misalignment reduction of the PICCS approach introduced by Chen *et al*. The experimental results show that the present PWLS-ndiTV approach can achieve significant gains over the existing similar methods in terms of different measure metrics. Furthermore, this study demonstrates that the present PWLS-ndiTV approach has useful potential for radiation dose reduction by reducing the projection data in the case of repeated CT scan performed in clinic.
